# Neurosensory Alterations in Retinopathy of Prematurity: A Window to Neurological Impairments Associated to Preterm Birth

**DOI:** 10.3390/biomedicines10071603

**Published:** 2022-07-06

**Authors:** Martina Lucchesi, Silvia Marracci, Rosario Amato, Luca Filippi, Maurizio Cammalleri, Massimo Dal Monte

**Affiliations:** 1 Department of Biology, University of Pisa, 56127 Pisa, Italy; martina.lucchesi@student.unisi.it (M.L.); silvia.marracci@unipi.it (S.M.); rosario.amato@biologia.unipi.it (R.A.); maurizio.cammalleri@unipi.it (M.C.); 2 Department of Clinical and Experimental Medicine, Division of Neonatology and NICU, University of Pisa, 56126 Pisa, Italy; luca.filippi@unipi.it

**Keywords:** neurodegeneration, photoreceptors, retinal ganglion cells, retinal glia

## Abstract

Retinopathy of prematurity (ROP) is one of the main blinding diseases affecting preterm newborns and is classically considered a vascular disorder. The premature exposure to the extrauterine environment, which is hyperoxic in respect to the intrauterine environment, triggers a cascade of events leading to retinal ischemia which, in turn, makes the retina hypoxic thus setting off angiogenic processes. However, many children with a history of ROP show persistent vision impairment, and there is evidence of an association between ROP and neurosensory disabilities. This is not surprising given the strict relationship between neuronal function and an adequate blood supply. In the present work, we revised literature data evidencing to what extent ROP can be considered a neurodegenerative disease, also taking advantage from data obtained in preclinical models of ROP. The involvement of different retinal cell populations in triggering the neuronal damage in ROP was described along with the neurological outcomes associated to ROP. The situation of ROP in Italy was assessed as well.

## 1. Introduction

In the latest decades, the survival rate of extremely preterm and low birth-weight infants has drastically improved [1]. Although this could represent an important achievement reflecting the advanced efficacy in the clinical management of preterm infants, it also increases the risk of short- or long-term complications deriving from preterm birth.

Among them, retinopathy of prematurity (ROP) is one of the main ocular disorders affecting preterm newborns that may result in a significant loss of vision or even blindness [2]. Extremely preterm infants are at high risk of developing ROP. In particular, about 50% of them show clinical signs of ROP, although this percentage may vary. In 2010, a study evaluated that over approximately 185,000 preterm infants were affected by ROP and more than 20,000 were affected by complete loss of visual function [3]. So far, the greatest risk predictors of ROP are a low gestational age and a low birth weight. In this respect, in countries with high-quality neonatal care, sight-threatening ROP is mainly confined to infants with a birth weight lower than 1000 g and is very rare in babies with a birth weight higher than 1250 g [4].

The common denominator of preterm birth complications, including ROP, is represented by drastic changes in physiochemical parameters due to the precocious passage from the intrauterine to external environment, which influences the development of immature tissues and organs. In the case of the retina, its organogenesis occurs relatively late in the gestational period. Therefore, the preterm birth drastically impairs the morphofunctional organization of the still immature organ, creating a wide range of structural and functional changes in both neural and vascular components. Since the early discovery of ROP, its pathogenesis has been mainly attributed to the change in oxygen tension when passing from a hypoxic condition, as in the intrauterine environment, to a relative hyperoxic condition, as in the extrauterine environment. 

**Figure 1:**
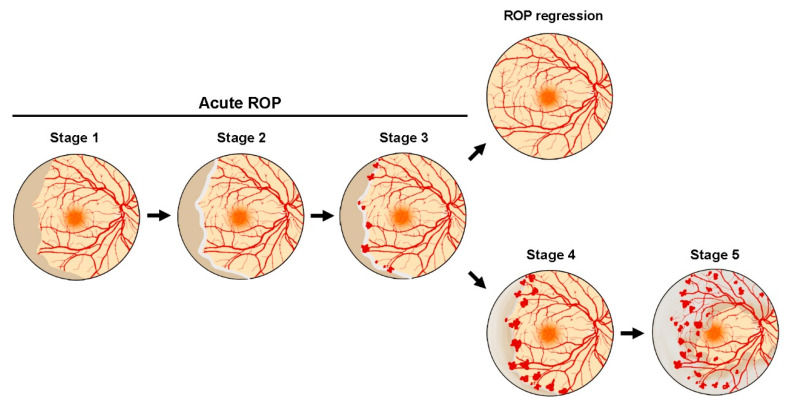
Schematic representation of retinopathy of prematurity (ROP) stages. Stage 1 is characterized by the appearance of a demarcation line between the vascular and the avascular zone of the retina, which may evolve in a visible ridge in stage 2. In stage 3, proliferating retinal vessels depart from the ridge to gradually occupy extraretinal spaces towards the vitreous. Stages 1–3 represent the acute phase of ROP, which could resolve in a spontaneous regression of the abnormal neovascularization or could further evolve in the more severe stages 4 and 5, characterized by partial and total retinal detachment, respectively.

The hypoxia physiologically present in the pregnant uterus represents the ideal environment where retinal vascularization of the fetus can develop [5]. The early retinal vasculature originates from spindle-shaped precursor endothelial cells, that migrate by 14–15 weeks of the postmenstrual age from the optic disk towards the ora serrata, and the early networks of capillaries are formed from 17–18 weeks of the postmenstrual age [5]. The relative hypoxia promotes the vascular development through the production of a dimeric nuclear transcription factor, the hypoxia inducible factor 1 (HIF-1), which modulates a plethora of oxygen-sensing genes including a series of proangiogenic factors, such as a vascular endothelial growth factor (VEGF) [6]. Under hypoxia, the subunit HIF-1α accumulates into the cell, migrates into the nucleus and, after dimerization with the subunit HIF-1β, activates the transcription of VEGF [7]. In the developing retina, which becomes physiologically hypoxic after the onset of neuronal activity, VEGF is mainly (but transiently) produced by neuroglial cells, to stimulate in a paracrine fashion growth, and migration of endothelial cells that organize to form the retinal vessel network [8]. The formation of a functional retinal circulation relieves the retina from hypoxia and, under oxygen exposure, HIF-1α subunit is hydroxylated, ubiquitinated and finally degraded, thus reducing the transcription of HIF-1 target genes [7]. Through such a mechanism the exposure of the immature retina to atmospheric oxygen leads to the interruption of the vascularization processes, which normally occur within the intrauterine hypoxic environment, thus leaving the peripheral retina avascular until an approximate gestational age of 32 weeks. The interruption or even the regression of the vascularization process establishes an ischemic condition (ischemic phase of ROP), which gradually worsens together with the increasing metabolic demand of the developing retina. The persistent ischemia and the deriving hypoxia drive the switch to the proliferative phase of ROP, which is characterized by the abnormal activation of angiogenic processes leading to the outgrowth of dysfunctional and disorganized new vessels. In addition to further exacerbating the ischemic condition, the proliferation of aberrant neovessels may also induce the development of intravitreal fibrosis with consequent retinal traction and detachment [9]. Considering that vascular abnormalities manifest the most pathological hallmarks in ROP, the location and the appearance of the vascular aberrations are currently considered as the main parameters for the classification of disease progression and severity from stage 1 to stage 5 (Figure 1). Stages 1–3 describe the acute phases of the disease characterized by the formation of a demarcation line between the vascular and the avascular retina (stage 1) that will evolve in a ridge (stage 2) from which extraretinal neovascular proliferation will arise towards the vitreous (stage 3). From the acute stages of ROP, vascular abnormalities can either spontaneously regress turning to a regular retinal vascularization, or further evolve in stages 4 and 5 defined as severe ROP, characterized by partial and total retinal detachment, respectively [10].

The importance of oxygen in the pathogenesis and in the progression of ROP has become evident with clinical trials demonstrating that limiting oxygen delivery to preterm newborns reduces the risk of the disease but increases their mortality [11]. Therefore, although the fine control of oxygen delivery to preterm infants is currently the first line of prevention of ROP progression, a saturation target lower than 90% is not acceptable, and the persistence of a certain ROP incidence is considered an unavoidable consequence of a reduced mortality [12]. Conversely, significant steps forward have been made in the characterization of the oxygen-dependent mechanisms contributing to the vascular abnormalities. 

Fundamental contributions in the study of ROP physiopathology have derived from the use of the in vivo model of oxygen-induced retinopathy (OIR) in rodents [13]. This model exploits the plasticity of retinal vessels typical of neonatal mice and rats, whose vascular plexa physiologically develop after birth [14]. The OIR model firstly consists of the exposure of pups to hyperoxia, which interferes with the process of retinal vascularization and induces a wide vaso-obliteration (around the optic nerve head in mice; in the peripheral retina in rats). Then, newborn pups are returned to a normoxic environment, with a sudden reduction in retinal oxygenation that is perceived as a relative hypoxia. As is the case of ROP, the ischemia/hypoxia in the OIR retina triggers a dramatic increment in angiogenesis causing abnormal vessel sprouts along with hemorrhages and vitreous edema due to the vascular hyperpermeability [13]. 

The primary players in the mechanisms linking the alterations in retinal oxygenation with the abnormal vascular sprout, in ROP as well as in the OIR model, are HIF-1 and VEGF. Indeed, the HIF-1-dependent dramatic downregulation of VEGF occurring under hyperoxia drives the interruption of vascularization and the vaso-obliteration, while VEGF overexpression under hypoxia is the leading mechanism causing vascular hyperpermeability and proliferation [7]. With VEGF having a relevant role in ROP pathogenesis, it has been identified as a useful target in treating ROP. In fact, cryotherapy or the less painful laser photocoagulation, for a long time, were used as the gold standard treatment for infants with severe ROP, aimed at destroying the hypoxic peripheral retina, thus reducing VEGF production [15]. However, this approach is burdened by serious adverse effects (need of anesthesia, non negligeable risk of repeated intervention, and visual dysfunction) [16] and complications (corneal burns, band keratopathy, cataract) [17]. The currently available alternative treatment that avoids retinal destruction is the intravitreal injection of neutralizing anti-VEGF drugs. In respect to laser photocoagulation, anti-VEGF drugs have been recently reported to display a decreased incidence of retinal detachment, probably thanks to a faster decrease in VEGF levels [18], a reduced rate of optic atrophy and amblyopia [19], and less eye complications even if burdened by a higher retreatment incidence [20]. However, the short- or long-term safety of anti-VEGF drugs still arouses some concern, and further investigations are required to evaluate their effects on retinal developmental processes [21].

## 2. ROP in Italy

The birth rate in Italy, calculated using the birth rate of 2020 [22], is approximately 410,000/year and 1% of these newborns are born with a birth weight lower than 1500 g or at a gestational age lower than 30 weeks, thus indicating that about 4000 infants are at risk for ROP. Borroni et al. [23] analyzed the incidence and the associated risk factors of ROP and aggressive posterior-ROP (AP-ROP), a severe form of ROP, in 25 Italian neonatal intensive care units through a prospective multicenter observational study examining 421 infants born with a birth weight lower than 750 g and/or a gestational age shorter than 27 weeks. Starting the ophthalmologic screening at about 30 weeks of gestational age, ROP was diagnosed in 62.9% of the preterm infants, while AP-ROP was diagnosed in 24.2% of them. At a univariate analysis, ROP was significantly associated with gestational age, body weight, intraventricular hemorrhage, patent ductus arteriosus, bronchopulmonary dysplasia, erythropoietin therapy and sepsis, while AP-ROP was significantly associated with gestational age, bronchopulmonary dysplasia, and sepsis. Performing a multivariate analysis, ROP was found to be associated only to intraventricular hemorrhage and erythropoietin therapy, while AP-ROP was found to be associated to gestational age and sepsis. The incidence data of this Italian study does not match with those of the CRYO-ROP and of the ETROP studies [24,25], possibly because these studies were performed before the introduction of the guidelines for ROP prevention [26], which were applied in the Italian study. On the other hand, the results of the work of Borroni et al. [23] are in agreement with those of other studies and of the Vermont–Oxford Network, which is a non-profit organization of health care professionals collaborating to improve neonatal care [27,28,29,30]. The finding that the use of erythropoietin may be associated with the development of ROP has been confirmed by another Italian study [31]. This study found that in preterm infants with a body weight lower than 1000 g receiving erythropoietin therapy, the occurrence of ROP was double than that of preterm infants that did not receive erythropoietin, suggesting this therapy as an independent risk factor for ROP development. More recently, the incidence and risk factors for ROP were examined in 2 Italian neonatal intensive care units, through a retrospective study involving 178 infants with a gestational age lower than 29 weeks [32]. This study found an occurrence of ROP of 38%, similar to that found in other studies [33,34,35,36,37,38,39,40], and an association between red blood cell transfusion and the risk of ROP. The finding that red blood cell transfusion may represent a risk factor for ROP development is in agreement with other studies [41,42,43,44,45,46]. However, red blood cell transfusion is often essential to treat anemia in preterm neonates (very common due to the prematurity itself and to repeated blood sampling). In this respect, a retrospective study involving neonates with a gestational age lower than 32 weeks demonstrated that the risk for developing ROP carried by blood products is likely to depend on the gestational age. In particular, the gestational age at the second transfusion seems to be even more useful to determine the risk for severe ROP development, outperforming all other variables in predicting severe ROP [47].

There are premature infants with apparent similar characteristics, sharing similar clinical features (gestational age, birth weight, oxygen support), who have a completely different ROP evolution, with some of them needing treatment, and others experiencing spontaneous regression of the pathology. Therefore, there is an urgent need of standardizing evaluation criteria and identifying early indicators for ROP development. In this respect, a recent retrospective Italian study reported that early platelet counts were significantly reduced in newborns who later developed severe ROP which required treatment, but not in those who never developed ROP [48,49] suggesting that platelet count may be used to detect in advance premature infants prone to ROP development. Recently, a new prediction model for ROP, the Postnatal Growth and ROP (G-ROP), which was originally developed in 2018 and validated in a North American cohort of preterm infants [50], has been also validated in Italy [51]. G-ROP is based on birth weight, gestational age and weight gain, and allows to reduce the number of infants undergoing ROP examinations by about 30% while maintaining 100% sensitivity for ROP when compared to the criteria currently in use, thus being very promising for future clinical adaptation. Its validation in the Italian population lays the foundation for the introduction of G-ROP criteria in all European countries.

If on the one hand, the list of ROP risk factors is becoming longer, on the other, a growing body of evidence is progressively highlighting the ability of a variety of compounds, both naturals and pharmacological, to prevent ROP or to reduce its progression towards later and more deleterious stages. For example, Garofoli et al. [52] demonstrated in a cohort of preterm infants with birth weight lower than 1500 g that orally administered vitamin A is effective in attenuating ROP incidence and severity. Moreover, Filippi et al. widely demonstrated that propranolol, a usually well-tolerated non-selective beta adrenoceptor antagonist is able to counteract the progression of ROP [53,54,55]. These clinical trials followed preclinical studies performed in OIR mice in which systemic or topic propranolol administered during the hypoxic phase of the disease reduced retinal neovascularization by preventing HIF-1α upregulation and its proangiogenic cascade [56,57].

Although in preterm infants oral propranolol is not sufficiently safe [54], propranolol 0.2% eye micro-drops showed an optimal safety and tolerability profile and efficiently reduced ROP progression [58,59]. It is noteworthy that propranolol seems to exert a neuroprotective effect in the OIR retina, suggesting that beta adrenoceptor blockade may be useful in human patients not only to prevent neovascularization but also to limit the neural damage evidenced by impaired ERG [60]. Indeed, OIR mice showed an altered ERG profile due to neuronal cell death, while the propranolol treatment protected retinal cells enhancing pro-survival pathways such as autophagy while inhibiting apoptosis, thus finally resulting in recovered retinal function [61]. However, clinical trials performed to date are not large enough to draw definitive conclusions about propranolol efficacy in treating ROP nor on its supposed neuroprotective effect [60]. In the meantime, the results obtained in ROP infants treated with propranolol so far, especially related to its antiangiogenic effects, are very promising.

## 3. The Impact of ROP in the Central Nervous System

Preterm children are at risk of developing poor visual outcomes with respect to children born at full term, independently to the presence of ROP. Indeed, there is a large cohort of studies indicating that a reduced visual acuity, defined as the capacity of the central visual system to discriminate contrast variation, can be associated to preterm-born children with or without ROP, with visual impairment lasting into adulthood. Therefore, this suggests that a poor visual outcome may be considered a long-term functional consequence of preterm birth [4,62,63,64,65,66]. On the other hand, ROP development in preterm infants impacts on visual outcomes, and acuity deficits in patients with a history of ROP are typical, even though the pathology has resolved completely [64]. For instance, a Swedish population-based prospective study conducted in children of 10 years who had been born preterm, found that visual impairments were present in 26% of those with no ROP and in 64% of those with severe ROP, while they occurred in 8% of controls born at term [67]. In the same line, a population-based prospective study, made in New Zealand on young adults born preterm before ROP treatment was available, reached the conclusion that adults who had developed ROP had reduced visual acuity when compared to adults with no ROP, indicating that ROP increases the risk of developing poor long-term visual outcomes [68]. In this respect, both the CRYO-ROP and the ET-ROP studies found a correlation between severity of acute-phase ROP and development of visual acuity [24,25]. Notably, an examination over a period of 18 years of adolescents and adult patients with previously regressed ROP showed a late reactivation of ocular deficits together with a reduced visual acuity, highlighting that a decreased visual acuity can occur as a long-term outcome in patients with resolved ROP [69]. Treatment for ROP became available after the publication of the results of the CRYO-ROP study [24] and there is evidence that treating ROP ameliorates visual outcomes, with anti-VEGF agents giving better results on visual acuity than laser photocoagulation. For instance, a study published by Rodriguez and colleagues in 2020 evaluated visual acuity in children aged over 4 years treated with anti-VEGF, comparing their visual acuity with that measured in the ET-ROP study published in 2010 [70]. This study revealed that 85% of eyes treated with anti-VEGF had normal visual acuity with respect to 35% of eyes receiving laser photocoagulation, suggesting that different ROP treatments may have different impacts on visual outcomes over the years [71], a conclusion shared with other works as, for instance, a recent study performed at the UCLA Medical Centers [19]. In this context, it would be important to clarify whether anti-VEGF may actually have greater effects over laser photocoagulation, in order to maximize preterm newborn visual outcomes, since they could affect, at least in part, the everyday activities in adult life.

Although ROP may be associated with later visual impairments, it may also impact on non-visual abilities. In fact, besides ocular injuries, infants with ROP may exhibit further neurodevelopmental impairments. Given the common embryonic derivation of eye and brain, it has been hypothesized that pathological processes associated to ROP (such as oxygen fluctuations, oxidative stress, inflammation) could also have harmful effects on the development of different regions of the central nervous system (CNS) [72,73,74].

Notably, cerebral cortex and cerebellum development occurs during the gestational third trimester and are vulnerable to detrimental environmental factors. Thus, the early exposition to extrauterine environments as well as damage to the vascular and neuronal pathways during maturation, could alter CNS development. As an example, ROP disease is characterized by the dysregulation of insulin-like growth factor 1 (IGF-1) and VEGF and it is known that both these factors play an important role in neurogenesis, neural differentiation, axon maturation, and neuroplasticity [75,76,77]. In particular, based on the fundamental roles of IGF-1 on neurodevelopment, it is possible to speculate that altered IGF-1 levels may affect the correct development of CNS regions. In line with these hypotheses, the postnatal decrease in circulating IGF-1 observed in very preterm infants has been found to associate with a low brain volume at term age, suggesting that normalizing IGF-1 levels may result in the neuroprotection of these patients [78]. Similarly, changes of VEGF levels may lead to improper CNS development. For instance, the use of intravitreal bevacizumab injections induces a risk of neurodevelopmental delay in ROP infants, suggesting that anti-VEGF therapies may introduce a risk of developmental impairments [79].

However, it remains to be determined whether ROP and any neurological alterations in the CNS may depend on a shared etiological origin and further studies will be necessary to elucidate this point. From another point of view and considering that the retina itself is an anatomical and functional portion of the CNS, all the interventions that may have an impact on ROP and reduce the risk factors associated to the disease may also have a beneficial effect on the development of those CNS structures altered as a consequence of premature birth. 

### 3.1. Neurological Outcomes Associated with ROP

Studies investigating possible associations between ROP and neurological outcomes are in progress. Presently, there is a general agreement that severe ROP may represent a risk factor for neurosensory impairments in childhood, although studies exploring possible connections between ROP severity and neurodevelopmental disorders have led to controversial conclusions. For instance, a study performed in extremely preterm infants with a gestational age lower than 26 weeks suggested that severe ROP is a good predictor for major neurosensory disabilities, including cerebral palsy, severe visual impairment, and hearing loss. In particular, preterm infants with severe ROP have been reported to display a significantly poorer outcome at 11 years than preterm infants without ROP, with neurosensory impairments detected in 50% of the infants who had suffered from severe ROP [80]. Similar conclusions have been reached in other studies relative to cohorts of premature children suffering from severe ROP evaluated at age 1.5 or 5 years [81,82,83]. In addition, in a Swedish study performed on 27 preterm children totally blind due to ROP stage 5, the authors found that 75% of them had major neurological impairments, including mental retardation, cerebral palsy and epilepsy, indicating an association between severe ROP and worsened neurodevelopment [84]. In this respect, at the beginning of this century, two studies aimed to assess the relationship between ROP and neurodevelopmental functions in the same cohort of preterm children, evaluated at 5.5 and 8 years, reached the conclusion that severe ROP may be considered a marker for functional disabilities developed in very low birth weight infants [85,86]. In the same period, a study enrolling 115 preterm infants, none of which were suffering from severe ROP, associated moderate ROP with worsened motor and cognitive disabilities, an association that, however, was found borderline after correction for gestational age [87]. In 2014, Allred et al. reported that preterm children with severe ROP followed up at 2 years corrected age (that is the chronological age minus the number of months the preterm infant is born early), showed a higher probability of impaired neurodevelopment characterized, for instance, by brain lesions and cerebral palsies [88]. In 2018, Drost et al. performed a study to compare the volumes of different brain structures and the cortical morphology of preterm infants with severe ROP with those of control infants with the same gestational age, birth weight and sex. This analysis, carried out using magnetic resonance imaging (MRI), indicated that preterm infants with severe ROP have smaller cerebellar volumes and reduced cortical gyrification. Cognitive and motor abilities at 15 months corrected age, were shown to be worse in infants with severe ROP than in controls [75]. In addition, a cohort study using MRI analysis carried out by Sveinsdóttir et al. in infants of 2 years corrected age, showed that infants with ROP had lower unmyelinated white matter and cerebellar volume as well as reduced mental and psychomotor developmental indices with respect to infants without ROP. However, infants with less severe ROP exhibited a reduction in brain volume and a neurodevelopmental impairment similar to those observed in infants with more severe ROP, suggesting that any stage of ROP may result in neurodevelopmental deficits and that preventing ROP, also in its less severe stages, may positively impact brain development [89]. In the same line, Glass et al. found that severe ROP is associated with white matter alterations and with a delayed maturation of brain regions involved in visual and motor processing [90], suggesting that severe ROP may be associated to neurodevelopmental outcomes. In an additional cohort study on ROP-diagnosed children, 68.4% of ROP infants showed neurodevelopmental disabilities, including blindness, cerebral palsy, cognitive, motor, speech and hearing problems. However, approximately half of ROP children with normal visual capacities exhibited neurodevelopmental disabilities, thus suggesting that preterm ROP infants have a high risk for developing neurodevelopmental anomalies even when their visual abilities are normal and underscoring the importance to prevent ROP not only to avoid blindness but also to hamper neurodevelopmental disorders [91].

Different from the studies discussed above, some other works suggest that neurodevelopmental alterations may be more strongly associated to preterm birth than to ROP. For instance, two studies comparing preterm infants with ROP stage 3 with those with ROP stage 2 and ROP stage 1/no ROP and evaluating their developmental outcome, found that preterm children in all groups have reduced neural development that was not associated with ROP severity, suggesting that neurodevelopmental disabilities are most likely associated with premature birth instead of ROP severity [92,93]. In 2007, Stephenson et al. performed a retrospective study on 505 subjects of 11–14 years that were born prematurely, of whom 49% had developed ROP, and assessing visual and cognitive outcomes. The study concluded that neither poor ophthalmic nor poor cognitive outcomes results were associated to previous stage 1 or stage 2 ROP, and that stage 3 ROP is associated to poor visual acuity but not to cognitive impairment, suggesting that unexplored factors in addition to ROP may explain the poor cognitive performance [94]. Ahn et al., using MRI, evaluated the microstructural integrity of brain white matter in preterm infants with and without ROP and in full-term infants. The results demonstrated alterations in 15–17 of the 23 predefined regions in which the brain was divided. Fewer differences were instead observed comparing preterm infants with and without ROP, limited to 2 of the 23 predefined regions, thus suggesting that alterations of white matter maturation could be associated with preterm birth rather than with ROP [95]. The same research group reached the same conclusions in a more recent work showing that ROP or severe ROP were not associated with white matter abnormalities in preterm infants of 18 months corrected age with or without ROP. Particularly, developmental outcomes, including cognitive, language and motor functions, were similar between preterm infants with or without ROP and independent from ROP severity [96]. In line with these studies, Altendahl et al. examined cognition, language, and motor scores of premature neonates at 0–36 months corrected age, screened for ROP, and found that ROP severity was not associated with worse neurodevelopmental scores [97]. Similar results were obtained in a recent study evaluating the presence of hearing loss in a large cohort of preterm infants at 18 months corrected age, revealing no significant associations between ROP and hearing impairments [98]. Overall, these studies suggest that negative neurodevelopmental outcomes in preterm neonates are plausibly related to prematurity-associated factors more than to ROP severity and that ROP, *per se*, does not seem to contribute significantly to neurodevelopmental diseases.

The controversial correlation between ROP (and, eventually, its severity) and neurodevelopmental outcomes made evident by the studies cited in the present paragraph, highlights the necessity of extensive studies investigating brain development and its alterations in infants suffering from ROP in order to reach more robust and accurate conclusions.

### 3.2. Neurodegeneration in the Retina of ROP Patients

**Figure 2:**
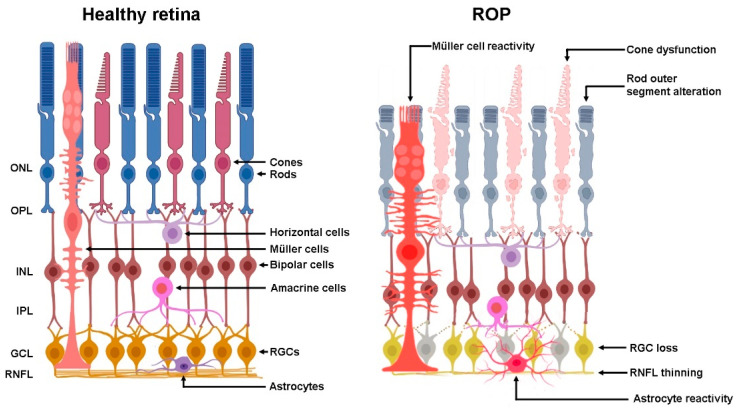
Schematic representation of neuroretinal alterations in ROP according to evidence from preterm infants and experimental model of oxygen-induced retinopathy. ONL, outer nuclear layer; OPL, outer plexiform layer; INL, inner nuclear layer; IPL, inner plexiform layer; GCL, ganglion cell layer; RNFL, retinal nerve fiber layer; RGCs, retinal ganglion cells.

From a clinical point of view, ROP is classified as a vascular disorder. In line with this, most literature focuses on the mechanisms leading to neovascularization and the current therapeutic treatments are directed to the normalization of the retinal vasculature [99]. However, despite the effectiveness of these treatments or the spontaneous regression of the pathology, many children with a history of ROP experience persistent vision impairment, such as astigmatism and myopia [100], reduced visual acuity and deficient subtle color vision [101], constricted visual field and contrast sensitivity [102], and increased dark-adapted thresholds [103], along with structural retinal abnormalities [4,104]. Overall, these data suggest that some form of alteration in the development, viability, and function of neuroretinal cells (photoreceptors, retinal neurons and glia), may occur during ROP progression (Figure 2). This is not surprising, given the existence of a strict functional relationship between neurons, glia and blood vessels known as neurovascular unit, in which chemical signals released by neurons and/or glial cells regulate the surrounding blood vessels to adequately support the metabolism of neuronal cells [105]. However, in retinopathies the relationships between vascular abnormalities and neurodegeneration can be of various types. For instance, in diabetic retinopathy, a retinal disease that develops years after the diagnosis of diabetes, neurodegeneration occurs early during disease development and, at least in part, precedes the vascular damage [106]. On the contrary, in ROP the disease progresses in a short time and the damage to the neural part of the retina is likely to be secondary to the development of vascular abnormalities.

Relevant insights about the neuroretinal alterations in ROP derive from functional analyses performed by means of electroretinography. An electroretinogram (ERG) is classically composed of a biphasic waveform displaying an early negative component (a-wave), elicited by the light-induced response of the photoreceptors, followed by a positive component (b-wave) generated by post receptor cells [107]. Depending on retinal light adaptation, the ERG may reflect the activity of rod-mediated (dark-adapted scotopic ERG) or cone-mediated (light-adapted photopic ERG) pathways. ERG analyses in patients with ROP or in animals with OIR have revealed significant functional abnormalities in both the inner and outer retina. In patients with ROP, ERG response results are significantly attenuated and are accompanied by impaired contrast sensitivity and decreased scotopic visual thresholds [13,100,108,109,110]. ERG deficits are also evident in OIR animals [111,112], in which both a- and b-wave alterations generally correlate with structural deficits. Although the attenuation of the a-wave in OIR animals has been reported to occur without the apparent loss of photoreceptors [113], it seems to be related with a thinner photoreceptor outer segment layer, which is more disorganized than in normal controls [114]. Animals with OIR also exhibit a reduced thickness of the inner plexiform layer (IPL) and of the inner nuclear layer (INL), with a concomitant increase of apoptosis in the inner retina [113]. These changes might affect the ERG due to loss of retinal neurons and disruption of synaptic transmission, which involve largely, but not exclusively, the regions of vascular dysfunction [115].

#### 3.2.1. Photoreceptors

The photoreceptors are the last cells to complete maturation, with the increase of outer segment elongation and the escalation of photopigment content occurring in the last 8 weeks of gestation. Therefore, alterations of the photoreceptor maturation due to the preterm birth might cover a relevant role in the onset of long-term visual dysfunctions in ROP [116].

##### Rods

Rods are the last retinal cell subtype to reach maturity, with their rhodopsin content in the outer segment appearing and increasing from approximately 32 weeks of gestational age [108,114]. Together with their maturation, the energy demand of rods rises due to the increased turnover of the light transduction-related molecules in the outer segment and to the increment in rod electrical activity. Alterations in oxygen supply due to a still incomplete and insufficient retinal vasculature, as it occurs in ROP, produce significant abnormalities in the maturation of rod outer segments. Indeed, they could be shorter, containing a low amount of rhodopsin and consequently showing diminished quantum catch, impaired mobility of the transduction cascade proteins in the disc membranes. Accordingly, infants affected by ROP display significant alterations in retinal activity demonstrated by the ERG analysis [110]. In this respect, although the abnormal ERG response may derive from an overall damage of the whole neuroretina, as demonstrated in the OIR model, strong evidence supports the possibility that alterations in the outer segments of rods may have a primary counterpart in the ERG dysfunction [114]. This is even more evident in parafoveal rods, in which the developmental elongation of outer segments is physiologically delayed compared to peripheral rods. Thus, rods in the late-maturing parafovea are more vulnerable to the effects of ROP. In fact, the development of rod-mediated scotopic threshold is prolonged in infants with ROP compared to controls, more so in the parafoveal than in the peripheral retina. The precise underlying mechanisms remain to be defined, but they could include a slower rate of rod outer segment elongation, delayed packing of parafoveal rods (along with the delay in the foveal pit—see the section below), or disorganization of rod outer segments [117].

##### Cones

Primate cone maturation occurs earlier than that of rod outer segments, thus implying a higher resistance to the alterations caused by premature birth [109]. In addition, cones appear to be more resistant to metabolic insults, having twice as many mitochondria and three times the surface area of mitochondrial cristae compared to rods [118]. However, a recent study [119] has underlined the possibility that, together with the rods, cones might also undergo some form of functional alteration that manifests years after birth. Using multifocal ERG (mfERG), which allows to concomitantly measure the light-adapted activity of cones and that of post receptor cells in different areas of the retina, a significant dysfunction of the cone pathway in the central retina, characterized by low-cone sensitivity and slower recovery of cone responses, has been reported in subjects with a history of severe ROP [119]. Importantly, the magnitude of mfERG alterations varied together with the severity of the antecedent ROP but, notably, did not completely match with the canonical categorization of ROP based on the vascular abnormalities, as the case of no differences in mfERG amplitudes were detected between children with a history of mild ROP and children with no ROP. Therefore, this would suggest that the current ROP categorization, exclusively based on the characteristics of the retinal vasculature at the time of examination in the nursery, is quite reductive, since it does not take into account the important effects observed in the neurosensory retina.

##### The Foveal Pit

Macula lutea development is a sophisticated process which is not completed until at least the third or fourth year of life; particularly, the fovea centralis is the last retinal structure to reach complete maturity and it mediates the excellent visual acuity in healthy adults [120,121]. Within the macula lutea, the foveal pit (or fovea centralis) is an avascular zone formed by the centrifugal displacement of inner nuclear cells and retinal ganglion cells (RGCs) toward the periphery [122].

ROP significantly delays foveal development [123]. It also alters the foveal structure, although data on this point are not univocal. Indeed, Hammer et al. [120] reported that the foveal pit of subjects with a history of spontaneously regressed ROP appears broad and shallow, while some years later Wang et al., studying a slightly wider group of subjects, observed a diminished depth and a reduced shallow slope of the ROP foveal pit compared to controls [124]. 

Certainly, ROP determines the presence of retinal capillaries intertwined with the neural cells that cover the fovea [120] and increases foveal thickness [121]. This latter outcome is mainly due to the presence of inner retinal layers overlying the fovea, probably due to a failure of the inner retinal neurons to migrate away from the pit [124]. In line with this, studies on preterm children with or without a history of ROP reported that the thickness of both the RGC layer (GCL) and the IPL was remarkably higher at fovea centralis compared with full-term controls, while no differences were observed at more peripheral retinal locations [124,125,126]. 

Wang et al. found that the increased foveal thickness did not result in any changes in visual acuity [124]. However, other studies demonstrated the existence of a correlation between inner retinal layer thickness and worsening of the best-corrected visual acuity (BCVA) in extremely preterm infants [127,128], while there was no correlation between altered BCVA and the thickness of outer retinal layers. Moreover, worsened BCVA is associated with an increased GCL thickness in extremely preterm infants, possibly as a consequence of the arrest of GCL maturation due to preterm birth, which prevents RGC death, a major event in retinal development [127].

#### 3.2.2. Retinal Ganglion Cells

RGCs are particularly sensitive to changing oxygen levels. In this respect, hypoxia has been established to severely impact RGC survival and RGC axon outgrowth [129,130]. The significant damage to RGCs in the developing retina under altered oxygenation has mainly been demonstrated in the OIR model, in which the hypoxic phase is primarily responsible for inducing RGC degeneration and subsequent decrement in RGC density [130]. In this model, the alteration in RGC survival has been established to depend on the imbalance between apoptosis and autophagy during the hypoxic phase, resulting in the degeneration not only of RGCs, but also of bipolar and amacrine cells [61]. In this respect, there is evidence from our group that treatments based on natural antioxidant/anti-inflammatory compounds may prevent RGC death in several models of RGC degeneration [131,132,133,134], suggesting that such an approach may be useful also in counteracting neurodegeneration of RGCs in ROP. The use of nutraceuticals as a non-invasive approach in preventing ROP and in its management has been recently reviewed and discussed [135].

Evidence about the altered neural pro-survival signalling also derives from preterm infants affected by severe ROP, which display serum levels of neurotrophin-4 and brain-derived neurotrophic factor (BDNF) during the first 3 weeks of life that are lower than those in preterm infants who did not develop severe ROP. In the same cohort of patients, specific gene variations of BDNF were associated with threshold ROP [136,137], indicating the possibility that a possible alteration in neural trophism could concur to RGC degeneration during ROP progression. Besides their overt loss, evidence of a significant RGC damage is represented by structural alterations of their axons within the retinal nerve fiber layer (RNFL).

Studies of optical coherence tomography or spectral-domain optical coherence tomography revealed that RNFL thickness is altered in premature newborns compared to full-term children. In this respect, the RNFL resulted in a greater thickness on the temporal side of the optic disc in preterm infants than in full-term controls, whereas all other RNFL sectors were thinner. Such alterations in RNFL thickness were correlated with gestational age and birth weight, two key factors related with the risk of ROP development [138,139,140], as well as with the stage of ROP progression [141].

Whether RNFL alterations would correlate with visual function is still under debate. In this respect, Park et al. found no association between RNFL thickness and visual function [141]. On the contrary, Wang et al. in an analysis of 25 preterm and 54 full-term infants, reported an association between RNFL thickness in the retinal temporal sector and visual acuity [139]. Similarly, Fieβ et al. demonstrated that decreased RNFL thickness in all retinal sectors is associated with reduced visual function in both preterm infants and full-term neonates, underscoring the high risk of preterm infants to develop alterations in the RNFL in association with a decrease in visual function [140].

#### 3.2.3. Müller Cells and Astrocytes

Müller cells are the main retinal glial cells. From their soma, located in the INL, two major projections extend towards the GCL and the photoreceptor layer. Processes generated from these projections reach and surround neurons, synapses, and blood vessels creating physical and chemical relationships that allow intimate communications of central importance for physiological retinal functions [142]. Among the functions Müller cells accomplish, they: i. provide neurons with trophic factors and antioxidants; ii. uptake and recycle neurotransmitters including glutamate, thus avoiding excitotoxic insults to neural cells; iii. transport water, ions and metabolites through channels and transporters participating, for instance, to K^+^ buffering and to the control of the composition of the retinal milieu; iv. participate to the formation of the blood-retinal barrier by secreting factors that enhance the barrier function of the surrounding endothelium [143]. 

Astrocytes, star-shaped glial cells mainly located in the GCL and in the RNFL, also play important roles in retinal physiology. For instance, astrocytes provide metabolic support to RGCs, thus controlling their ionic and metabolic homeostasis, and regulate neurovascular coupling. They also have a paramount role in the development of retinal vasculature; astrocytes are indeed the earliest glial cell type in the optic nerve of the embryo and, migrating into the neuroretina and releasing proangiogenic factors including VEGF, critically contribute to the formation of retinal blood vessels. They also act as a scaffold on which retinal vessels develop from the centre of the retina to the periphery. In this respect, astrocytes are lacking within the avascular foveal pit and are present only in the vascularized retina, a finding that highlights astrocyte importance in the development of retinal vasculature. In addition to inducing the formation of retinal blood vessels, similarly to Müller cells astrocytes contribute to the formation and maintenance of the blood retinal barrier [144].

When retinal homeostasis is challenged, glial cells undergo morphological and functional changes (gliosis or glial activation) with the attempt to avoid conditions potentially leading to a diseased state; however, gliosis may have not only beneficial but also detrimental effects on retinal function [145]. In Müller cells, the upregulation of proteins contributing to the formation of intermediate filaments, including glial fibrillary acidic protein (GFAP), is a hallmark of gliosis; in fact, in the healthy retina GFAP is only minimally expressed by Müller cells, which, on the contrary, dramatically increase GFAP expression in retinal diseases [146]. After their activation, glial cells secrete trophic factors to support neuronal and vascular function; however, if gliosis becomes chronic, direct and indirect damage to neurons and vessels may occur. For instance, the overproduction of VEGF leads to blood retinal barrier leakage, while the release of inflammatory cytokines may result in neuronal degeneration [147,148,149].

In humans, massive retinal gliosis is a rare, benign, intraocular condition that develops in association with other ocular diseases and is mainly constituted by Müller cell activation [150]. There are only a couple, not very recent, case reports describing the association between massive retinal gliosis and ROP [151,152]. One of them reported the presence of bilateral massive gliosis in a 39-year-old man born prematurely that developed severe ROP with retinal detachment [151], suggesting that severe ROP may have deleterious effects during the patient life. Although no clinical evidence indicates that gliosis may participate in ROP pathogenesis, results in preclinical models suggest this possibility. In fact, it has been demonstrated that gliosis, and in particular Müller cell activation, is a feature of OIR rodents [13] and that in these animals, maneuvers attenuating Müller cell gliosis reduce retinal neurovascular degeneration and preserve retinal function [153,154,155].

## 4. Future Perspectives

**Figure 3:**
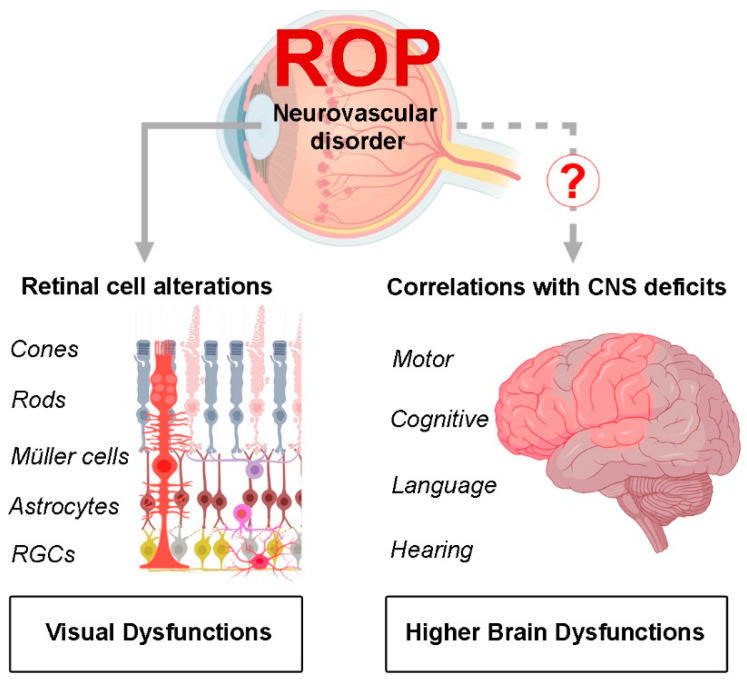
ROP, as neurovascular disorder, leads to retinal cell alterations finally resulting in visual dysfunctions. Further studies are required to evaluate whether long-term neurological outcomes observed in patients that had developed ROP are actually related to the disease or are instead a consequence of premature birth. CNS, central nervous system.

Although ROP has long been considered a vascular disorder, there is evidence raising the question on how this disease may be linked to neurodegenerative processes that may involve higher brain functions (Figure 3). Therefore, future studies will be necessary to investigate whether long-term neurodevelopmental outcomes can be considered associated to functional alterations characterizing ROP pathology. The involvement of the neural components in ROP, and not only of the vascular ones, should be taken into account when considering the resulting retinal structural and visual abnormalities. Such an approach could identify new targets for interventions in order to give children affected by ROP the best possible visual outcome.

## Data Availability

Not applicable.
